# Evaluation of the Preservation Value and Location of Farm Ponds in Yunlin County, Taiwan

**DOI:** 10.3390/ijerph110100548

**Published:** 2013-12-31

**Authors:** Wen-Wen Chou, Soen-Han Lee, Chen-Fa Wu

**Affiliations:** 1 Department of Architecture, National Cheng Kung University, Tainan City 701, Taiwan; E-Mails: hotto8@msn.com (W.-W.C.); sunhan2010@hotmail.com.tw (S.-H.L.); 2 Department of Horticulture, National Chung Hsing University, Taichung City 402, Taiwan

**Keywords:** farm pond values, preservation location selection, decision support

## Abstract

Farm ponds in Yunlin County first appeared in 1,622 and have played roles in habitation, production, the ecology, culture, and disaster reduction. Farm ponds largely disappeared with the development of urban areas and the industrial sector; thus, effective preservation of the remaining ponds is critical. The criteria to evaluate the preservation value of farm ponds is established by expert questionnaires which follow the Fuzzy Delphi Method (FDM) and Fuzzy Analytic Hierarchy Process (FAHP), and GIS, which are integrated into a spatial analysis of the remaining 481 farm ponds in Yunlin County. The results show that 28 ponds should be preserved to continue the cultural interaction between farm ponds and settlements; 36 ponds should preserved to connect coasts and streams, which are important habitats for birds; 30 ponds should be preserved to increase storage capacity, recharge groundwater, and reduce land subsidence; four ponds should be preserved as Feng-Shui ponds in front of temples in settlements or as recreation areas for local citizens; and four farms should be preserved (high priority) in agricultural production areas to support irrigation. In short, FAHP and GIS are integrated to evaluate the number and locations of farm ponds that provide water for habitation, production, the ecology, culture, and disaster reduction and maintain the overall preservation value in Yunlin County. The results could inform governmental departments when considering conservation policies.

## 1. Introduction

The diversity and role of farm ponds have been emphasized in the past few years. Scheffer *et al*. noted the remarkable biodiversity benefits of small-scale wetlands or pools [1] and artificial alternatives such as farm ponds [2], which provide habitats for aquatic animal and plant species [3]. The focus on pool conservation could benefit the preservation of local amphibians [4,5,6]. In research on *Gallinula chloropus guami* in the Territory of Guam, Ritter *et al*. found that 80% of the amphibian population would select artificial wetlands, serving as pools for hydroponic crops, water storage, cattle rearing, and golf courses, as a habitat in either the dry or wet season. Such pools with aquatic plants would function as a habitat for nesting and hunting [7]. Casas *et al*. studied the types and physicochemical characteristics of farm pond wetlands and natural wetlands in southern Spain to evaluate the structure, hydrology, chemical characteristics, and latent importance to determine if farm ponds can act as a substitute for aquatic habitats to preserve biodiversity [8]. The results showed that among agricultural landscapes, small-scale artificial water habitats preserved aquatic diversity in distinct climates, and numerous farm ponds could serve as habitats for numerous and diverse shorebirds. Research in the UK indicated that historical ponds were able to preserve more local species, and various types of ponds could support the habitats of different wildlife during development [9]. In addition, aquatic plants in farm ponds can mitigate metal pollution [10]. The Pond Protection Alliance and Pond Life Project were established in the UK, showing the importance of protecting and developing ecological environments and preserving the natural and cultural resources represented by European farm ponds [11].

Farm ponds assist in reducing the effects of environmental disasters, with streams, ponds, irrigation channels, and paddy fields acting as micro-reservoirs to store water and delay the peak time of flood surface runoff. Camnasio *et al*. indicated that flood detention pools such as farm ponds could provide agricultural water in environments suffering from water shortages and reduce summertime drought [12]. For this reason, effectively integrating streams, ponds, irrigation channels, and paddy fields into a micro-reservoir system could increase water storage and delay surface runoff speed [13]. Sun *et al*. used ASTER remote sensing images to investigate the urban cooling island (UCI) effect of lakes and five rivers in Beijing. The UCI intensity was related to temperature differences and gradient changes between the water body and the surrounding landscape. The results also showed the significant effect that the water body’s area and shape had on the cooling effects of urban areas, with wetland areas correlating positively with the distance of land-surface temperature changes and correlating negatively with the temperature change and gradient. In addition, numerous small-area water bodies were more effective than larger water bodies of the same total area [14]. Water bodies such as farm ponds reflect the diversity between culture and nature and the indirect relationships between human habitats and complex natural environments and combine cultural, social, and natural environments to create new water bodies that reinvent the original landscapes [15]. In addition, farm ponds could be used for local ecological or environmental education [16,17]. Therefore, farm ponds present multiple benefits to agricultural production, social and cultural values, ecological conservation, and environmental preservation [18]. Farm ponds are not simply pools for agricultural irrigation but are multi-dimensional water bodies that support industry, culture, the economy, and the ecology and mitigate environmental disasters. 

A total of 2,276 farm ponds were found in a land-use digital map created by the National Land Surveying and Mapping Center in 2007. The objective of this study is to evaluate the preservation value of farm ponds and the preserved objects. The Fuzzy Delphi Method (FDM) and Fuzzy Analytic Hierarchy Process (FAHP) are commonly integrated to obtain evaluation criteria for determining the value of environmental resources [19,20,21]. In this work, the criteria to evaluate the preservation value of farm ponds were established by FDM and FAHP, and GIS, which were integrated into a spatial analysis of the locations for farm pond preservation in Yunlin County, Taiwan.

## 2. Material and Methodology

### 2.1. Study Area and Farm Ponds

Taiwan has an area of approximately 3.6 million hectares, a maximum altitude of 3,952 m above sea level and an average annual rainfall of approximately 1,500 mm. There is an uneven distribution of rainfall throughout the year, with the wet season (May to October) receiving approximately 80% of the annual rainfall, which rapidly drains into the ocean without being conserved for agriculture; approximately 20% of annual rainfall occurs during the dry season (November to April) and is not enough for agricultural production. To cope with the uneven rainfall and insufficient irrigation systems, farmers began digging farm ponds for agricultural use [22]. A total of 42 ponds were built for water conservation in the Yunlin area during the Ching Dynasty (1684–1895) [23,24]. During the period of Japanese rule (1896–1949), the Chianan irrigation system represented the most significant construction for water transportation. Pumping groundwater or digging farm ponds for water was utilized for irrigation until Chianan irrigation system completed. Recently, rapid urban development, roadway construction, and increasing population growth and construction in rural areas have facilitated the disappearance of farm ponds. The number of farm ponds in Yunlin County dropped from 2,921 in 1995 down to 2,276 in 2007, representing approximately 22.08% of farm ponds. Currently, the preservation of the remaining farm ponds is considered critical.

**Figure 1:**
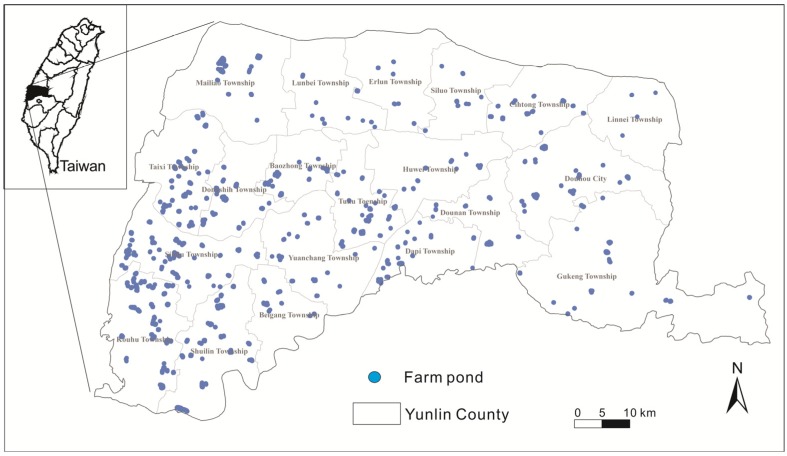
Research areas and location of farm ponds.

Investigating the remaining 2,276 farm ponds in Yunlin County requires significant funding and time. Farm ponds located in cultivated areas are generally utilized for agricultural and aquacultural production. In this study, we try to identify farm ponds that are located in non-agricultural areas, such as rural valleys, to identify multifuntions of farm ponds. The following principles are applied to the research subject selection: the settlement can contain more than 50 households within a 500 m radius of a farm pond and farm ponds used purely for professional aquaculture are excluded. A total of 481 ponds are selected, and the location distribution is shown in Figure 1.

### 2.2. Methodology

The Delphi method, proposed by Dalkey and Helmer in 1960, is a systematic method of integrating a group of expert opinions. Murray first introduced the fuzzy concept into the Delphi Method [25], and Ishikawa and Amagasa *et al*. utilized Dual Triangular Fuzzy Number in a Fuzzy Integration of expert opinions, which is named the Fuzzy Delphi Method (FDM) [26]. Cheng modified the method to rapidly integrate expert questionnaires and reduce the number of repeated questionnaires [27]. Because the Fuzzy Delphi Method can reduce investigation time, accurately express the opinions of experts, and show economic benefits in time and cost, it is utilized for the evaluation of farm pond preservation values with the operating processes listed below.

Establishing evaluation criteria and collecting the opinions of experts is the first step in determining the preservation value of farm ponds. Based on a theoretical analysis of farm pond preservation value, a questionnaire was created (Table 1) for 26 experts: two in rural rejuvenation, four in landscape management, five in ecological conservation, three in wetland conservation, three in cultural landscape management, three in architecture, and six in other fields. The expert consensus with Fuzzy Delphi Method is used for confirmation (Table 1). The Fuzzy Delphi Method is then used for screening evaluation factors. Each expert gives a possible interval value for each evaluation criterion, where the minimum interval value is “the most conservative cognition” of the quantified criteria and the maximum is “the most optimistic cognition” [28]. Each evaluation item i is calculated as “the most conservative cognition (*C^i^_L_*)” and “the most optimistic cognition (*C^i^_U_*)” of the experts. The most extreme value beyond “twice the standard deviation” is removed for calculating the minimum *C^i^_L_*, the geometric mean *C^i^_M_*, and the maximum *C^i^_U_* of “the most conservative cognition” and the minimum *O^i^_L_*, the geometric mean *O^i^_M_*, and the maximum *O^i^_U_* of “the most optimistic cognition”.

Testing the expert consensus consists of six steps. The first step does not contain gray areas and is represented by *C^i^_U_* ≤ *O^i^_U_*, two non-overlapping triangular fuzzy numbers in which each expert’s interval value appears as the consensus interval and the opinions approach the consensus interval. The “consensus importance” of the evaluation criterion *G^i^* is represented as *G^i^* = (*C^i^_M_* + *O^i^_M_*)/2. The second step contains gray areas representing a small opinion gap among the experts. The triangular fuzzy numbers are overlapped, which is represented as *C^i^_U_* > *O^i^_L_*, and the gray area of the fuzzy relation is represented as *Z^i^* = *C^i^_U_* − *O^i^_L_* < *M^i^* = *O^i^_M_* − *C^i^_M_*, revealing that the interval value of the experts’ opinions does not represent the consensus interval but that the opinions are not divergent. The “consensus importance” of the evaluation criterion i is represented as *G^i^* = *min* (fuzzy relations of two triangular fuzzy numbers), and the fuzzy set is further calculated according to the quantified mean of maximum. The third step contains gray areas and a large gap in consensus among experts. The two triangular fuzzy numbers appear to be overlapping (*C^i^_U_* > *O^i^_L_*), and the gray area of the fuzzy relations is represented as *Z^i^* = *C^i^_U_* − *O^i^_L_* > *M^i^* = *O^i^_M_* − *C^i^_M_*, which shows the interval value of each expert’s opinion that does not appear in the consensus interval and the divergence of opinions based on significant differences between opinions with extreme values and other opinions. The fourth step involves testing the evaluations of the hierarchical factors, deleting the factors that have differing opinions, and establishing the evaluation structure. The fifth step involves calculating the hierarchical weights. The evaluation structure established by screening the factors is further calculated by the mean of the hierarchical weights and relative weights, including the “optimum value” of expert consensus. The sixth step is the actual investigation of the farm ponds. A total of 481 farm ponds were investigated within 12 months, from 1 May 2010 to 31 April 2011, and at least two researchers investigated each pond for four hours. The locations and areas of the farm ponds were acquired from the 2007 GIS map data from the National Land Surveying and Mapping Center in Taiwan; the water yield and water sources of the farm ponds and the current use of the surrounding land were measured on site; and information regarding the formation, management, and maintenance of the farm ponds was acquired by interviewing the neighborhood magistrates or owners of the farm ponds. 

When the test value is Z*^i^* > 0, the experts’ opinions are consistent and the evaluation criteria achieve convergence in the Fuzzy Delphi Method. Higher Ci values reveal a higher expert consensus and importance; there is no standard threshold because the threshold is generally judged by research objectives and subjective opinions. Additional factors are deleted at higher thresholds, and at lower thresholds, more factors are preserved. Factors are calculated by multiplying the arithmetic mean (7.14) of the expert consensus (*G^i^*) by 0.9 to acquire the integral 6.65 as the threshold; factors that fall beneath this threshold are removed. The results of the analyses show that the test value of the expert consensus in Hierarchy I (“Value of Agricultural Production”, “Value of Civic Life”, “Value of Ecological Attributes”, “Value of Environmental Disaster Reduction”, and “Value of Cultural Landscapes”) is greater than 0, which shows the experts’ opinions approaching consistency and an expert consensus above 6.65 (all factors are preserved) (Table 1). Of the 16 evaluation criteria in Hierarchy II (Table 1), the only test value less than 0 with an expert consensus (6.05) lower than the threshold involved connecting clan affection in the settlements, which was removed. The remaining criteria have test values greater than 0 and expert consensuses above 6.65 (preserved), representing a consensus of expert opinion. From the results, the evaluation structure and criteria of farm pond preservation values are confirmed. Criteria in Hierarchy I include the dimensions of Value of Agricultural Production, Value of Civic Life, Value of Ecological Attributes, Value of Environmental Disaster Reduction, and Value of Cultural Landscapes and the criteria in Hierarchy II covers 15 factors (Table 1). After screening and confirmation of the criteria with the Fuzzy Delphi Method, the geometric mean of the optimum value of the experts’ cognition is used to calculate the relative weights. The relative weight, importance, and order of the criteria in various hierarchies are shown in Table 1.

**Table 1:** Weights of preservation value evaluation of farm ponds.

Objective	Hierarchy I criteria		Hierarchy II criteria		Total weight A × B	Order	Reference
	Evaluation criteria	Weight (A)	Evaluation criteria	weight (B)			
**Farm ponds Preservation Value**	A. Value of Agricultural Production	0.196	A.1	0.102	19.99 × 10^−3^	3	[10,11,29]
			Agriculture Irrigation				
			A.2	0.094	18.42 × 10^−3^	4	[24,30,31]
			Aquaculture				
	B. Value of Civic Life	0.197	B.1	0.049	9.65 × 10^−3^	13	[18,24]
			Ponds exemplifying traditional rural life				
			B.2	0.047	9.26 × 10^−3^	14	[13,32]
			Recreational green space in communities				
			B.3	0.053	10.44 × 10^−3^	9	[11,30]
			Evidence of historical development				
			B.4	0.047	9.26 × 10^−3^	14	[18,24]
			Feng-shui ponds of settlements or temples				
	C. Value of Ecological Attributes	0.204	C.1	0.050	10.20 × 10^−3^	11	[3,4,33,34,35]
			Diverse habitats for animals				
			C.2	0.050	10.20 × 10^−3^	11	[3,21]
			Preservation of plant diversity				
			C.3	0.052	10.61 × 10^−3^	8	[2,4,21,32,36,37,38,39]
			Important inland wetland system				
			C.4	0.051	10.40 × 10^−3^	10	[10,40,41]
			Healthy aquatic ecological network				
	E. Value of Environmental Disaster Reduction	0.197	E.1	0.064	12.61 × 10^−3^	7	[14,42,43]
			Temperature reduction				
			E.2	0.067	13.20 × 10^−3^	5	[12,13,44,45]
			Flood detention				
			E.3	0.066	13.00 × 10^−3^	6	[46,47]
			Groundwater recharge for land subsidence				
	F. Value of Cultural Landscapes	0.206	F.1	0.102	21.01 × 10^−3^	2	[15,30,33]
			Witness of aged historical changes				
			F.2	0.104	21.42 × 10^−3^	1	[11,48,49,50]
			Continuous landscapes, presenting the spatial field of interaction between citizens and farm ponds				

Note: Connecting clan affection in settlements with the test value less than 0 is removed.

## 3. Results

### 3.1. Establishing the Evaluation Structure of Farm Pond Preservation Value

Analysis of the significance of criteria for Hierarchy I shows that the Value of Cultural Landscapes (0.206) has the highest weight (Table 1) followed by Value of Ecological Attributes (0.204). There is very little difference between the values, which reveals that preserving farm ponds is valuable because of their ability to extend local humanistic and historical culture and improve ecological attributes. The Value of Civic Life and Value of Environmental Disaster Reduction are weighted (0.197) and are almost equal to the Value of Agricultural Production (0.196), illustrating the similar importance of these preservation values.

Evaluation of hierarchy II includes a total of 15 factors: two in the Value of Agricultural Production, four in the Value of Civic Life, four in the Value of Ecological Attributes, five in the Value of Environmental Disaster Reduction, and two in the Value of Cultural Landscapes. Continuous Landscapes (0.104) has the highest relative weight, followed by Evidence of Historical Change (0.102), Agriculture Irrigation (0.102), and Aquaculture (0.094). These evaluation criteria are included under the covered in Value of Agricultural Production and Value of Cultural Landscapes in Hierarchy I, representing a high level of agreement among experts with respect to the historical humanistic value of farm ponds and the value of agricultural production. It also reveals that the importance of landscape changes in farm ponds after social and economic changes conforms to the new role and new orientation at the time of the change.

Flood detention (0.067), Temperature reduction (0.064), and Groundwater recharge for land subsidence (0.066) are the criteria contained in the Value of Environmental Disaster Reduction from Hierarchy I, showing the importance of expert consensus regarding Environmental Disaster Reduction. The weights of the remaining factors in the Value of Civic Life and the Value of Ecological Attributes in Hierarchy I include Important inland wetland systems (0.052), Preservation of plant diversity (0.050), Preservation of plant diversity (0.050), and Healthy aquatic ecological network (0.051) and have similar relative weights, showing that the ecological value of farm ponds is diverse and balanced when the value has been emphasized and there is a consistent perception of the pond’s ecological functions. Presenting the characteristics of traditional rural life (0.049), Recreational green space in communities (0.047), and Feng-shui ponds of settlements or temples (0.047) are the evaluation criteria for the Value of Civic Life and show low correlations between farm ponds and the environment of the local inhabitant’s daily life; thus, these criteria were of comparatively low importance in the evaluation structure.

Regarding the overall weights of the evaluation factors, the order of importance is as follows: a historical and continuous landscapes, agriculture irrigation, aquaculture, and flood detention. This result indicates that the value of cultural landscapes and the value of agricultural production are the key indicators in farm pond preservation. The value of environmental disaster reduction is also higher than most of the other evaluation criteria. Recreational green space in communities, Feng-shui ponds in settlements or temples, and Ponds exemplifying traditional rural life have the lowest weights among the evaluation factors under the Value of Civic Life, revealing that the Value of Civic Life does not equal the preservation value of farm ponds.

### 3.2. Preservation Value Evaluation of Farm Ponds in Yunlin County

The 2011 investigation data of the 481 farm ponds in Yunlin County are evaluated according to the evaluation structure of farm pond preservation value. The 15 evaluation criteria in Hierarchy II contain five levels and are scored (Table 2) according to the on-site investigation of farm ponds, with a higher level and score revealing a higher farm pond preservation value.

### 3.3. Location Analysis of Farm Pond Preservation Value

Farm pond preservation value was calculated by weighting the score of the evaluation criteria and summing scores to attain the total preservation value, which helped define the most important farm ponds to preserve. A standardization formula was use to define the farm ponds with high, middle, and low preservation value as follows:

D = (A − B) × C

H = A − D

L = B − D

A: Maximum preservation criterion of farm ponds in Hierarchy I
B: Minimum preservation criterion of farm ponds in Hierarchy I
C: Screening ratio C = 0.1, 0.2, or 0.3
D: Threshold of farm pond preservation evaluation in Hierarchy I
H: Farm ponds with high preservation value
L: Farm ponds with low preservation value

When C is set to 0.1, there are a low number of farm ponds with high preservation values in production, life, ecology, and overall preservation value (Table 3), but when C is set to 0.3, there are a high number of farm ponds with low preservation values in production and life. Because of this situation, locating farm ponds that have high, medium, or low preservation value is difficult. Nevertheless, the numerical distribution of high, medium, and low preservation values is still consistent with actual situations (Table 3). The results show the numbers of farm ponds with production, life, ecology, environmental disaster reduction, cultural landscapes, and overall preservation values at 4, 4, 36, 30, 86, and 16, respectively.

#### 3.3.1. Location Analyses of Farm Ponds with a High Preservation Value of Production

**Figure 2:**
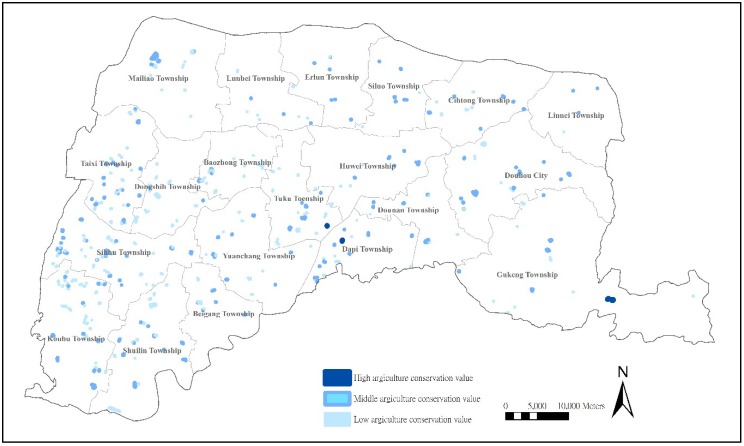
Distribution of farm ponds with production preservation values.

Farm ponds reveal characteristics of agricultural ecology and geographic landscapes [51]. In addition to the basic role of agricultural production, fish in these ponds were once a major source of animal protein for local human populations and enhanced the diversity of agricultural production [31]. From the evaluation of production value and the analysis of spatial location (Figure 2), the production value of farm ponds in Yunlin County is not high, with only four (0.83%) farm ponds presenting a high Value of Agricultural Production, but up to 298 (62.0%) presenting low production values. This result shows that the production value of most farm ponds in Yunlin County has been greatly reduced.

**Table 2:** Evolution criteria of farm pond preservation.

Evaluation criteria		Score = 1		Score = 2		Score = 3		Score = 4		Score = 5	
		Explanation	n	Explanation	n	Explanation	n	Explanation	n	Explanation	n
Agriculture Irrigation	Condition 1	1. Dry period of farm ponds > 3 months	465 (96.67%)	1. Dry period of farm ponds = 1–3 months	1 (0.21%)	1. Dry period of farm ponds <1 month	2 (0.41%)	1. Farm ponds with water year round	7 (1.46%)	1. Farm ponds with water year round and connected with waterways	6 (1.25%)
		2. Surrounding farms <50%		2. Surrounding farms <50%		2. Surrounding farms <50%		2. Surrounding farms <50%		2. Surrounding farms >50%	
		3. Without irrigation		3. With irrigation		3. With irrigation		3. With irrigation		3. With irrigation	
	Condition 2	None		1. Dry period of farm ponds > 3 months		1. Dry period of farm ponds = 1–3 months		1. Dry period of farm ponds <1 month		1. Farm ponds with water from underground springs year round	
				2. Surrounding farms >50%		2. Surrounding farms >50%		2. Surrounding farms >50%		2. Surrounding farms >50%	
				3. With irrigation		3. With irrigation		3. With irrigation		3. With irrigation	
Aquaculture		1. Water shortage year round	57 (11.85%)	1. Farm ponds with water	246 (51.14%)	1. Farm ponds with water	125 (25.99%)	1. Farm ponds with water	44 (9.15%)	1. Farm ponds lent out for aquaculture	9 (1.87%)
		2. No fish		2. Not managed		2. Bred and managed by community citizens		2. Privately managed		2. Breeding high economic crops such as crabs	
		3. Not managed		3. Natural growth of fish		3. Citizens sharing fish		3. Owners breeding fish			
Ponds exemplifying traditional rural life		1. Distance of farm ponds from settlements more than 50 m	132 (27.44%)	1. Distance of farm ponds from settlements within 50 m	75 (15.59%)	1. Distance of farm ponds from settlements within 50 m	150 (31.19%)	1. Distance of farm ponds from settlements within 50 m	89 (18.50%)	1. Distance of farm ponds from settlements within 50 m	35 (7.28%)
				2. No temples, buildings, leisure space, or five camps surrounding farm ponds		2. Temples, buildings, leisure space, and five camps surrounding farm ponds		2. Temples, buildings, leisure space, and five camps surrounding farm ponds		2. Temples, buildings, leisure space, and five camps surrounding farm ponds	
Recreational green space in communities	Condition 1	1. No environmental greenery	210 (43.66%)	1. Environment greenery <25% area around pond	189 (39.29%)	1. Environment greenery <75% area around pond	42 (8.73%)	1. Environment greenery >75% area around pond	26 (5.41%)	1. Environment greenery >75% area around pond	14 (2.91%)
		2. No leisure facility		2. No leisure facility		2. No leisure facility		2. No leisure facility		2. With leisure facilities	
	Condition 2	None		None		1. Simple green beautification		1. Medium green beautification		None	
						2. With leisure facilities		2. With leisure facilities			
Evidence of historical development		1. Not public ponds	261 (54.26%)	1. Not public ponds	73 (15.18%)	1. Not public ponds of settlements	58 (12.05%)	1. Public ponds of settlements	78 (16.22%)	1. Public pondsof settlements	11 (2.29%)
				2. Forming characteristics		2. Forming characteristics		2. Forming characteristics		2. Forming characteristics	
		2. No specific forming factor		2.1. Making adobes with the dirt		2.1. Making adobes with the dirt		2.1. Making adobes with the dirt		2.1 Making adobes with the dirt	
				2.2. Deposition of old rivers		2.2. Deposition of old rivers		2.2. Deposition ofold rivers		2.2 Deposition of old rivers	
		3. No specific stories or legends		2.3. Naturally formed in low areas		2.3. Naturally formed in low areas		2.3. Naturally formed in low areas		2.3 Natural formed in low areas	
				3. With specific forming stories or legends		3. With specific forming stories or legends		3. With specific forming stories or legends		3. With specific forming stories or legends	
Feng-shui ponds of settlements or temples		1. Not resident Feng-shui ponds of settlements or temples	450 (93.55%)	1. Feng-shui ponds merely for residence	1 (0.21%)	1. Feng-shui ponds merely for temples or settlements	16 (3.33%)	1. Feng-shui ponds merely for temples or settlements	13 (2.70%)	1. Feng-shui ponds for settlements and temples, presenting a feng-shui pattern	1 (0.21%)
Diverse habitats for animals		1. Farm ponds with water all year round, dry period >3 months	24 (4.99%)	1. Dry period of farm ponds 1–3 months	73 (15.18%)	1. Farm ponds with water, surrounding farms <50%	233 (48.44%)	1. Farm ponds with water, surrounding farms >50%	148 (30.77%)	1. Farm ponds with water, surrounding farms >50% connected with waterways	3 (0.62%)
Preservation of plant diversity		1. No plant surrounding farm ponds	70 (14.55%)	1. Low vegetation such as ground cover plants, shrubs, and trees	126 (26.20%)	1. Ground cover plants, shrubs, or trees with a higher level	216 (44.90%)	1. Ground cover plants, shrubs, or trees with 2 higher level	62 (12.89%)	1. Ground cover plants, shrubs, or trees with higher vegetation	7 (1.46%)
Important inland wetland system		1. Single farm ponds	27 (5.61%)	1. Single farm ponds	69 (14.34%)	1. Single farm ponds	315 (65.49%)	1. Dual farm ponds connected	69 (14.35%)	1. multi farm ponds connecte4d, with water all year round	1 (0.21%)
		2. Dry period >3 months		2. Dry period = 1–3 months		2. With water all year round		2. With water all year round			
Healthy aquatic ecological network	Condition 1	1. Single farm ponds not connected with other water system	409 (85.03%)	1. Farm ponds (2–3) without water, but not with water system	70 (14.55%)	1. More than 3 farm ponds, without connected waterways	2 (0.42%)	1. More than 3 farm ponds connected with waterways	0	1. More than 3 farm ponds connecting with waterways and rivers	0
	Condition 2	None		2. Single farm ponds, connecting with surrounding waterways or river systems		2. Farm ponds (2–3) connected with surrounding waterways or river systems		None		None	
Temperature reduction		1. No water all year round, or dry period 1–3 months	77 (16.00%)	1. Water all year round, farm ponds <0.25 hectare	135 (28.07%)	1. Area <0.25 hectare	43 (8.94%)	1. Area >0.25 hectare	124 (25.78%)	1. Area >0.25 hectare	102 (21.21%)
				2. Green belts, and paddy fields surrounding farm pond less than (equal to) 50%		2. Green belts and paddy fields surrounding farm ponds larger than 50%		2. Green belts and paddy fields surrounding farm ponds less than (equal to) 50%		2. Green belts and paddy fields surrounding farm ponds larger than 50%	
Flood detention	Condition 1	1. Not located in easily flooded areas	12 (2.49%)	1. Area <0.25 hectare, and located in floodplains in coastal areas, easily flooded areas, potential floodplains, or 100-year floodplains (one of above)	217 (45.11%)	1. Area <0.25 hectare, and located in floodplains in coastal areas, easily flooded areas, potential floodplains, or 100-year floodplains (two of above)	232 (48.24%)	1. Area >0.25 hectare, and located in coastal areas, easily flooded areas, potential floodplains, or 100-year floodplains (two of above)	20 (4.16%)	1. Area >0.25 hectare	0
										2. Located in coastal areas, easily flooded areas, potential floodplains, or 100-year floodplains (three of above)	
	Condition 2	None		None		2. Area >0.25 hectare, and located in floodplains in coastal areas, easily flooded areas, potential floodplains, or 100-year floodplains (one of above)		None		None	
Groundwater recharge for land subsidence	Condition 1	1. Not located in unconfined aquifer, funded areas with pressured water layer, and in land subsidence areas	21 (4.37%)	1. Area <0.25 hectare and located in unconfined aquifer, funded areas with pressured water layer, or in land subsidence areas (one of above)	192 (39.92%)	1. Area <0.25 hectare and located in unconfined aquifer, funded areas with pressured water layer, or in land subsidence areas (two of above)	246 (51.14%)	1. Area >0.25 hectare and located in unconfined aquifer, funded areas with pressured water layer, or in land subsidence areas (two of above)	1 (0.21%)	1. Area >0.25 hectare and located in unconfined aquifer, funded areas with pressured water layer, in land subsidence areas, or 3 km along high speed railway (two of above)	21 (4.36%)
										2. Several farm ponds connected	
	Condition 2					1. Area > 0.25 hectare and located in unconfined aquifer, funded areas with pressured water layer, or in land subsidence areas (one of above)					
Evidence of historical change		1. Newly dug (after 2007)	10 (2.08%)	1. Douliou Drain start (1957–2007)	183 (38.04%)	1. Douliou Drain completion in 1957 (1949–1957)	45 (9.36%)	1. Period of Japanese rule (1895–1949)	118 (24.53%)	Ching Dynasty (before 1895)	125 (25.99%)
Continuous Landscapes		1. Without original landscapes and changed the use being not water space	81 (16.84%)	1.Remaining landscapes of farm ponds, but with weak relationships with citizen memories and interaction	108 (22.45)	1. Maintain the use of farm ponds over time but changed to other uses in recent years. Water landscapes still apparent	129 (26.81%)	1. Landscapes of farm ponds have been largely changed but still remain in the water landscapes.	113 (23.50%)	1. The original use styles and landscapes remain.	50 (10.40%)
		2. Without relationship with citizen memories and interaction				2. Connection with citizens memories and interactions		2. Still interact with citizens and clearly remembered		2. Importance of farm ponds is greatly connected with citizen memories and interactions	

Note; Total number of pond is 481.

**Table 3:** Farm ponds preservation level evaluation.

	C:Gap 0.1 Threshold				C:Gap 0.2 Threshold				C:Gap 0.3 Threshold			
	High	N	Low	N	High	N	Low	N	High	N	Low	N
Preservation Value of Production	0.16196	**1**	0.0528	**57**	0.1483	**4**	0.0665	**298**	0.13468	**12**	0.08012	**414**
Preservation Value of Life	0.16232	**2**	0.0529	**132**	0.1486	**4**	0.0666	**195**	0.13496	**17**	0.08024	**319**
Preservation Value of Ecological Preservation	0.14086	**8**	0.0517	**8**	0.1297	**36**	0.0629	**22**	0.11858	**79**	0.07402	**45**
Preservation Value of Environmental Disaster Reduction	0.15712	**21**	0.0525	**1**	0.144	**30**	0.0656	**3**	0.13096	**149**	0.07864	**43**
Preservation Value of Cultural Landscapes	0.18952	**28**	0.0577	**5**	0.173	**86**	0.0742	**63**	0.15656	**156**	0.09064	**127**
Overall Preservation Value	0.67934	**3**	0.3061	**14**	0.6327	**16**	0.3527	**47**	0.58602	**50**	0.39938	**94**

The reduced production value is greatly affected by the acquisition water from irrigation and regional industrial transformation. The completion of the Douliou Drain in 1947 provided an important water source for agricultural production in Yunlin County, which reduced the importance of farm pond storage irrigation. The same situation is reflected in agricultural production areas in the central plains, revealing that our results are consistent with other research analyses. In addition, coastal industries have adopted aquaculture with higher economic value such that farm ponds have gradually disappeared or become the pools for aquaculture. Such a situation is common along the southwestern coasts and is consistent with research analyses.

#### 3.3.2. Location Analysis of Farm Ponds with High Preservation Values of Civic Life

**Figure 3:**
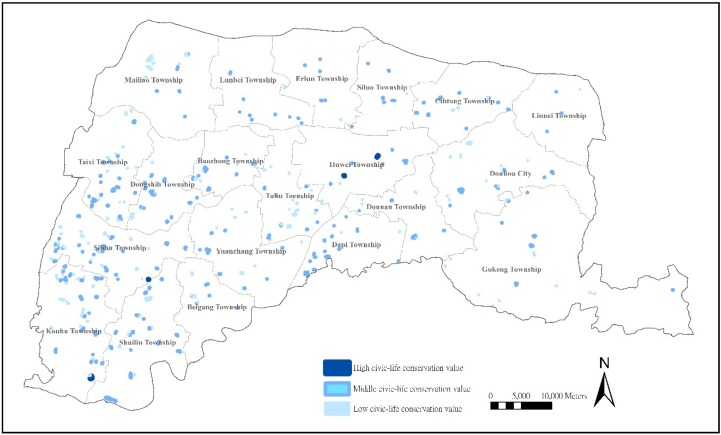
Distribution of farm ponds with civic life preservation values.

Farm ponds represent interactional areas for local inhabitants, social organizations, and natural environments [13], and the Value of Civic Life of farm ponds could best represent how farm ponds relate to regional landscapes and settlement development. Our research shows that only four (0.83%) farm ponds have a higher Value of Civic Life, but up to 195 (40.5%) reveal a low Value of Civic Life, with these low value farms mostly distributed along the western coasts (Kouhu Township, Sihhu Township, Taixi Township, and Mailiao Township), central plains (Tuku Township and Baozhong Township), and Douliou City. The distribution illustrates the polarization between cities and countryside (Figure 3).

The low value factors in the Value of Civic Life are closely related to the sustainable reuse and role changes of farm ponds. In the countryside along the eastern coasts (Kouhu Township, Sihhu Township, Taixi Township, and Mailiao Township), an aging population, bad living situations in settlements, and depressed traditional agriculture have resulted in farm ponds surrounded by pollution and garbage. The farm ponds are not managed and maintained, which detracts from the utility of the ponds reduces their value to civic life. Furthermore, farm ponds in the central plains (Tuku Township and Baozhong Township) and Douliou City have been transformed because of urbanization, industrialization, and social and economic changes [41]. With population growth, immigrants have settled in areas around farm ponds and they do not understand the historical and cultural meanings of farm ponds, which reduce their value to civic life. Meanwhile, the demand for land has resulted in farm ponds being incorporated into land for buildings, which often are not properly managed and maintained. For security reasons, fences are built around the farm pond, which further separates local citizens from the farm ponds.

#### 3.3.3. Location Analysis of Farm Ponds with High Ecological Preservation Values

In agricultural landscapes, farm ponds can be considered an alternative wetland habitat, with the water and buffer space preserving the value of aquatic diversity [8]. The size of farm ponds can improve biodiversity, provide unique species, and present significant contributions compared with other water bodies [38]. As a result, preserving farm ponds could actually benefit the preservation of ecological habitats and increase biodiversity.

**Figure 4:**
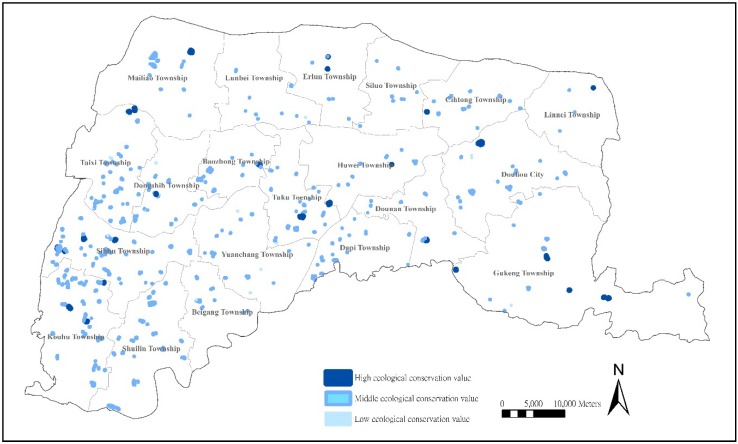
Distribution of farm ponds with ecological preservation values.

A total of 36 farm ponds (7.4% of total farm ponds), primarily distributed in coastal areas and eastern mountains but with several in the central plains, were shown to have a high ecological preservation value. Aquaculture was once a major industry on the southwestern coasts of Yunlin County, and many farm ponds were transformed into fish-breeding pools (Figure 4). 

The decline of aquaculture and outward migration in the recent years has resulted in numerous idle farm ponds, which then become favorable habitats for aquatic animals and establish coastal wetland habitats along coasts and at river mouths. The favorable natural environments in the eastern mountains connect farm ponds with the surrounding environment, and farm ponds become favorable habitats. Inland farm ponds with high Ecological Preservation Value maintain wide and fixed water connections with other farm ponds, rivers, and waterways and are established aquatic habitats, showing that the ecological environment preservation of inland farm ponds connected with water systems should be prioritized. 

Spatial locations show that 22 (4.57%) farm ponds with low ecological preservation value are concentrated on central inland plains and western inlands. Farm ponds in such areas encounter stricter environments, such as urbanization and insufficient water supplies, and agricultural reform and infrastructural development have resulted in the disappearance of biodiversity [3]. Therefore, suitable habitats cannot be created because the value of ecological attributes is comparatively low.

#### 3.3.4. Location Analysis of Farm Ponds with a High Value of Environmental Disaster Reduction

The environmental disaster reduction value of farm ponds lies in temperature reduction, flood detention, and groundwater recharge. Regarding the cooling effects of farm pond wetlands, numerous small wetlands present a more obvious cooling effect [14]. Farm ponds function in water storage, regional flood detention, and groundwater recharge when water resources are unevenly distributed [52]. As a result, widespread farm ponds contribute to regional temperature reduction, groundwater recharge, and regional flood detention.

**Figure 5:**
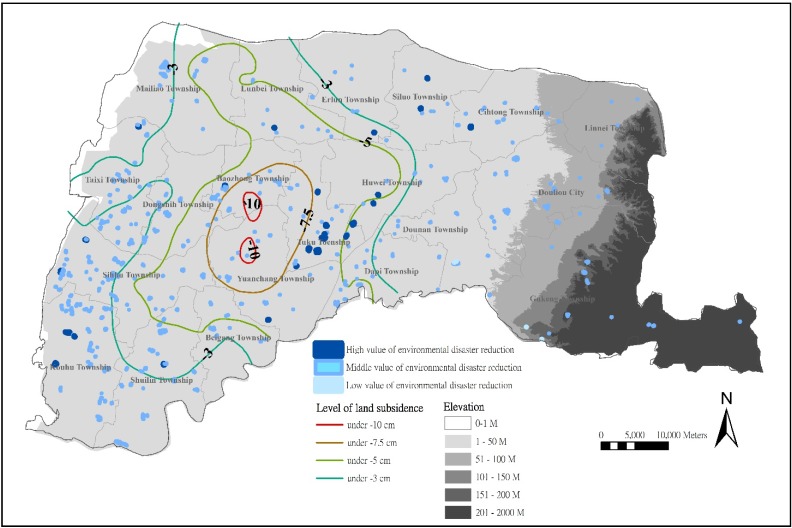
Distribution of farm ponds with preservation values of environmental disaster reduction.

Accordingly, an evaluation of preservation value could illustrate the value of environmental disaster reduction of farm ponds in Yunlin County. The evaluation shows that 30 (6.23%) farm ponds have a high value of environmental disaster reduction, and most of them are located in the central plains (Figure 5). 

Farm ponds in Tuku Township, Huwei Township, Siluo Township, Kouhu Township and Sihhu Township exhibit the cooling effects of small water bodies. Farm ponds, irrigation channels, and paddy fields can be considered micro-reservoirs, which can increase water yield, delay surface runoff speed, act as flood detention pools, and provide groundwater recharge. Inland farm ponds in Yunlin present a high potential for environmental disaster reduction (Figure 5). Only four (0.62%) farm ponds, located in the eastern hills, reveal the lower values of environmental disaster reduction (Figure 5). Farm ponds are located at higher altitudes; thus, they function in water storage but not flood detention.

#### 3.3.5. Location Analysis of Farm Ponds with High Value of Cultural Landscapes

Water is necessary for living and for activities that take place in the routine and non-routine centers of human populations. Therefore, water bodies could represent the diversity between culture and nature and the relationships between human habits and nature [15]. The natural water areas of farm ponds overlap with areas of human habitation and production such that the cultural landscapes of farm ponds give context to local history, culture, and ecological societies [11]. Early farm ponds were small pools next to farms and buildings designed for the agricultural economy and gradually developed into different types of farm ponds, thus showing their historical importance [9]. The overall analysis of regional development and settlement changes to the cultural landscape could illustrate the value that farm ponds add to cultural landscapes. According to our research structure, cultural landscapes illustrate the integration of farm ponds, the development of settlements, and interaction between local populations and farm ponds.

**Figure 6:**
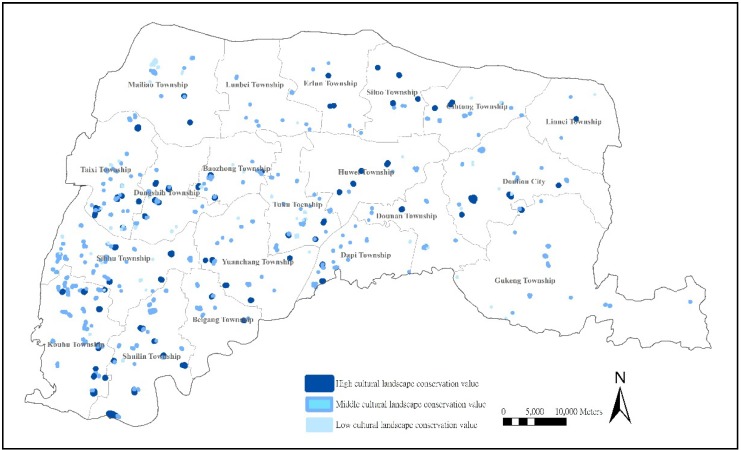
Distribution of farm ponds with cultural landscapes preservation value.

To determine the location and distribution of the cultural landscapes of farm ponds, the previous threshold is applied to the 86 (17.8%) farm ponds with high preservation value of cultural landscapes. As shown by the location distribution, most farm ponds with a high value of cultural landscapes are distributed along the southwestern coasts (including Kouhu Township, Shuilin Township, Beigang Township, Yuanchang Township, and Sihhu Township), central plains (Taixi Township and Dongshih Township), and central and northern areas (Huwei Township, Erlun Township, and Siluo Township) with several in the eastern mountains. Most are not concentrated in any one region, showing that farm ponds in Yunlin County extend humanistic environments and reflect the regional relationship between farm ponds and the spatial distribution of settlements (Figure 6). For instance, the Hutzunei settlement in Shuilin Township is the first farm-pond-centered spatial distribution. The settlement attaches to the natural hills and faces the farm pond; tall trees and a temple are the important spaces for village activities, presenting a spatial distribution corresponding to the environmental terrain. A total of 63 (13.1%) farm ponds with lower Cultural Landscape value are concentrated on the western coasts (including Sihhu Township, Taixi Township, and Dongshih Township), Mailiao Township, and inland plains (southern Tuku Township), showing a fractured interaction between farm ponds and human settlements and alienation from historical culture.

#### 3.3.6. Location Analysis of Farm Ponds with High Overall Preservation Value

The overall preservation value of farm ponds represents the integrated performance of cultural landscapes, civic life, agricultural production, environmental disaster reduction, and ecological conservation. The evaluation of overall preservation value could increase the understanding of diversity and importance of farm ponds in Yunlin County.

**Figure 7:**
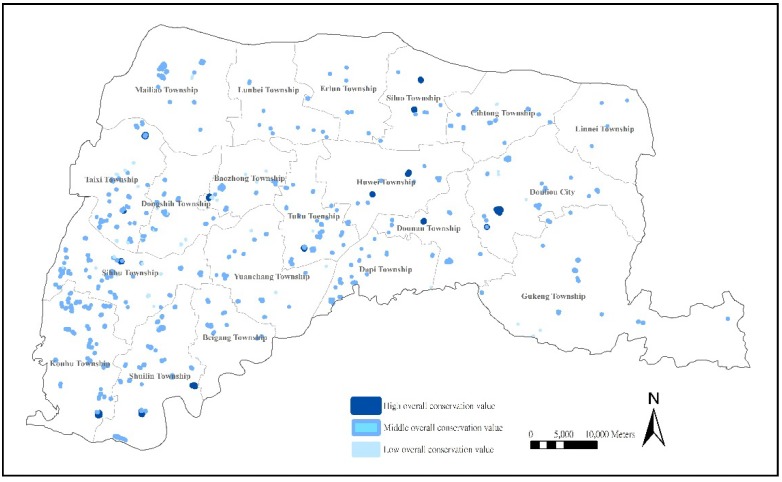
Distribution of farm ponds with overall preservation value.

The research shows that 16 (3.33%) farm ponds, which are mainly distributed in the central plains and the juncture of the southern Beikang River with a few along the western coasts, have a favorable overall preservation value, with farm ponds in Baochung, Huwei, and Dounan showing the highest overall preservation value, thus indicating that they are worth preserving. The 49 (10.2%) farm ponds concentrated in Taixi Township, Dongshih Township, and Sihhu Township along the western coasts have a low overall preservation value, revealing a high potential for improvement (Figure 7).

## 4. Discussion

### 4.1. Importance of Evaluation Criteria for Farm Pond Preservation Value

The research shows that the preservation values of farm ponds are divided according to the Value of Agricultural Production, Value of Civic Life, Value of Ecological Attributes, Value of Environmental Disaster Reduction, and Value of Cultural Landscapes. The Value of Cultural Landscapes has the highest weight (0.206), followed by Value of Ecological Attributes (0.204), Value of Environmental Disaster Reduction (0.197) while Value of Civic Life (0.197), and the Value of Agricultural Production shows the lowest weight (0.196). The evaluation criteria for calculating the farm pond preservation value reveals that the Value of Cultural Landscapes is highest and the Value of Agricultural Production is lowest. The criteria also show that farm ponds have changed from their original role in agricultural production irrigation because of the modernization of economic environments, which forces farm ponds to evolve complex and diverse relationships with local cultures, human habits, and nature, as farm ponds become new water bodies in old landscapes. Understanding the value of farm ponds and cultural landscapes could increase the value, meaning, and importance of culture in relation to natural landscapes, which are the characteristics of cultural landscapes.

Domestic and international research [3] indicate that farm ponds could offer habitats for various aquatic plants and species that they are the critical aquatic habitats in regional landscapes, which helps maintain biodiversity in regional environments [1]. The evaluation criteria for the preservation value of farm ponds also illustrates the importance of the ecological preservation value, as important inland wetland systems possess the most significant weight, showing that local farm ponds help preserve regional biodiversity. Preserving farm ponds could benefit the health of regional ecological environments and conserve local species. Farm ponds with a high Value of Environmental Disaster Reduction are increasingly important due to global environmental changes, permanent land subsidence in Yunlin County, flooding caused by severe climates in coastal areas [46], and reduced governmental funding for flood prevention engineering and flood disasters [44]. Locating farm ponds according to their water storage function can reduce regional temperatures; provide temporary flood detention, which reduces flood damage; and recharge the groundwater in land subsidence areas.

Regarding the rural-urban social-environmental divide, the combination of settlement development and farm pond preservation increases the Value of Civic Life and the quality of life in communities. As a result, the aquatic environments and green spaces on the banks of farm ponds could become an important leisure space in communities [53,54]. The close relationship between settlement development and water resources make farm ponds a primary element in settlements, which adds value to aspects of civil life that revolve around temples and the development of settlements.

### 4.2. Evaluation of Farm Pond Preservation Value and Location in Yunlin County

In addition to being a water source for irrigation and protein from fish in the pools, farm ponds are important in providing diversity in agricultural production. Farm ponds are characteristic of the agricultural ecology and geographic landscapes [51] and are thus numerous in Yunlin County. Nevertheless, the development of the Douliou Drain for irrigation and the fact that most farm ponds are not connected with waterways and rivers has resulted in their reduced value. The preservation values of the 481 farm ponds in Yunlin County show that only 4 farm ponds present high values to agricultural production, illustrating the disappearing function of farm ponds. Such a situation is obvious in coastal areas where agricultural industries are changing to aquaculture.

Research has shown the ecological value of farm ponds. In comparison with other water bodies, farm ponds show obvious contributions to local biodiversity [38] and could serve as a substitute for wetland habitats [8] in connection with an agricultural ecological network of surrounding farmlands, waterways, and river systems [11]. However, concrete farm ponds and surrounding facilities have resulted in changes to the agricultural ecological environment [55]. The ecological attributes of numerous farm ponds in Yunlin County are valuable, with 36 farm ponds having a high value of ecological attributes distributed along the southwestern coasts and eastern hills. Farm ponds in coastal areas represent large water bodies connected with coasts and river wetlands, forming sea-front aquatic habitats. Farm ponds in the eastern hills generate excellent habitats within the surrounding natural environment. In comparison, farm ponds in the central plains and inlands show a low value of ecological attributes because of urbanization, unstable water sources, and concrete farm pond structures.

Because of subsidence in important lands and expanding land subsidence areas in Taiwan [56], the environmental disaster reduction value of farm ponds must be prioritized. Small-scale farm ponds reduce surface runoff during heavy rains, reduce or release flood waters downstream [47], and recharge surface water sources [46]. A total of 30 farm ponds in Yunlin County, which are mainly distributed in the central plains and southwest coastal areas, reveal a high Value of Environmental Disaster Reduction. Farm ponds located in the central plains can store water for irrigation when water sources are not evenly distributed and become water sources that artificially recharge groundwater, enhance cooling effects, and reduce temperature. Farm ponds in coastal areas could be used in flood detention and help reduce the effects of flood disasters. Only four farm ponds show a low value of environmental disaster reduction in this study, and up to 447 (92.93%) farm ponds present medium values of environmental disaster reduction. If these ponds are maintained, their inherent environmental disaster reduction value could be reinforced by connecting them with waterways to reduce the effects of environmental disasters.

In early agricultural environments, local inhabitants maintained close relationships with water bodies. After societal and economical changes, the connection between farm ponds and the life of local human populations was greatly reduced and the dependence on farm ponds decreased [29]. Our research findings show that only 4 farm ponds have a high value of civic life, while 195 ponds have a low value of civic life. Human populations are distributed in polarized locations; urban areas consist primarily of immigrants who do not have a culturally significant connection with farm ponds. In urban areas, farm ponds present a safety risk to the surrounding population. The lack of water and leisure functions causes farm ponds to have a low value to civic life. In rural areas, an aging population results in insufficient management and maintenance of farm ponds, which reduce their value to civic life.

However, farm ponds have gradually become areas of increased cultural value. Farm ponds in Taiwan, in particular, are located in areas with diverse local people, living styles and skills, and social organizations [31] and show past and present value. Farm ponds in cultural landscapes are features that present outstanding universal value [50]. Our research shows that up to 86 farm ponds have a high value of cultural landscapes and are distributed along the southwestern coasts and central and northern plains. Cultural habits and legends regarding farm ponds have been maintained, and the recorded history, culture, and ecology allow a better understanding of the meaning and cultural value.

## 5. Conclusions

With expert questionnaires and the Fuzzy Delphi Method, the evaluation structure of farm pond preservation value is established and the evaluation criteria are calculated using relative weights. Cultural landscapes are the primary reason for farm pond preservation, and the provision of ecological environments for animals and plants is also important. Evaluation of environmental disaster reduction reveals that farm ponds help alleviate environmental changes, and the evaluation of civic life shows that farm ponds can provide benefits to local populations in addition to their traditional value in agricultural production. Spatial analysis in GIS found that the preservation of farm ponds in Yunlin County can be divided into three blocks. Farm ponds in southwest coastal areas primarily benefit life, ecology, and cultural landscapes, ponds in the central plains are more valuable in environmental disaster reduction and preservation of life, and ponds in the northeast are important to environmental disaster reduction. In sum, FDM, FAHP and GIS are integrated in this study to evaluate the number and locations of farm ponds with respect to habitation, production, the ecology, culture, disaster reduction, and overall preservation value. The results can be used to inform various governmental departments planning relevant conservation policies.
